# Effect of Holidays on National Vigor

**Published:** 1919-04

**Authors:** 


### EFFECT OF HOLIDAYS ON NATIONAL VIGOR

The revolutionary changes that the necessities of the war have injected into our national habits and conventional practices, have already become the subject of much speculation with respect to their future states. One often hears the query: "Shall we ever return to our former ways?" Particularly true is this in relation to the educational system, notably among the higher institutions of learning, which have responded in a most extraordinary manner to the nation's call for young men trained intensively in special ways to meet war-time emergencies. There has arisen a concentration of effort and a speeding up of schedules utterly unprecedented in the history of such establishments.

We should not be understood to imply that the outcome of this new way of conducting the business of education necessarily represents a beneficent reform. Undertaken to meet an emergency, it has demonstrated the capacity of our institutions to undergo rapid changes and adjustments of plan and policy. Even before any critical evaluation of the results achieved and the possible advantages gained can be attempted, we hear the prophet declaring that this country shall never again revive the slow and easy methods of the past. The catchword "efficiency"

is made to work overtime as the keynote of the expected performances of the future. We are warned that the mistakes of the past must not be repeated; that concentration of attention and conservation of the precious days and years are to become standard aims in the reconstruction period, alike in the world of education and in the domain of commerce.

More than one who have observed the actual working out of the new "militarized" procedures in practice, have seriously asked themselves whether in the long run such war-time measures can secure peace-time benefactions. The fact that every hour of the day is utilized does not guarantee that it is most advantageously employed. There is such a thing as intellectual indigestion resulting from inordinate concentration. The efficiency of a mechanical or automatic performance can doubtless be increased in large measure by persistently continued practice; on the other hand, there are more distinctly intellectual processes which become impaired unless a reasonable period for reflection and mental recuperation is allowed.

Let us hesitate, therefore, lest we adopt a machine-like scheme too rashly as the basis for the further development of American higher education. The physician has a special concern in the threatened abolition of the institution of holidays. To him who watches the mode of life of his fellow citizens, the beneficence of an occasional holiday has not escaped notice. It need not be debated whether there has been inordinate waste of time in the past; whether the life of many of our citizens, young and old, has not been extravagant in authorized idleness. The institution of suitable holiday periods is for the most part more than likely to make for good. "The right use of a holiday is one of the sovereign secrets in the practice of the noble art of keeping alive." Let us bear this in mind in the discussion of the projects of reconstruction, frankly admitting that leisure is valuable in the long run only if it improves the quality of work. A change of work may become a holiday in essence. The best holiday is not one spent in languid idleness but one that contains the largest amount of new experience. The physician needs such holidays; and in selecting their routine he will do well to observe such holiday experts as the naturalist, the traveler and the historian, rather than the golfer. A holiday, said a capable observer, is not an end in itself, but a means to a general improvement of the working life. The test of whether a holiday has or has not been well used, he added, is the supplement of zest and vigor that it contributes to our proper labors.—*Journal A. M. A.*, March 1, 1919.

